# Lycopene in Feed as Antioxidant and Immuno-Modulator Improves Broiler Chicken's Performance under Heat-Stress Conditions

**DOI:** 10.1155/2023/5418081

**Published:** 2023-06-29

**Authors:** Dalila Fadhila Hidayat, Mohamad Yusril Nur Mahendra, Juriah Kamaludeen, Herinda Pertiwi

**Affiliations:** ^1^Department of Health, Faculty of Vocational Studies Airlangga University, Surabaya, Indonesia; ^2^Department of Animal Science and Fishery, University Putra Malaysia, Bintulu Serawak Campus, Nyabau Road 97008, Serawak, Malaysia; ^3^Institute of Tropical Agriculture and Food Security, Universiti Putra Malaysia, Serdang 43400, Selangor, Malaysia

## Abstract

Lycopene is a type of carotenoid pigment widely distributed in various plants and fruits, with tomatoes, carrots, and guava being the most abundant sources. Due to its high content of beneficial active components, lycopene has been used in medicine, where it is employed as a dietary additive for cancer therapy, immune modulator, and feed additive to improve livestock productivity. Lycopene is a lipophilic substance that can act as either a prooxidant or a free radical scavenger and is particularly efficient in enhancing broiler performance. Furthermore, lycopene can alleviate heat stress by improving the activity of various antioxidant enzymes such as superoxide dismutase (SOD), glutathione peroxidase (GSH-Px), and catalase (CAT), as well as increasing the total antioxidant capacity (T-AOC) and nuclear muscle factor erythroid 2-related factor 2 (Nrf2), while simultaneously reducing the levels of malondialdehyde (MDA) and muscle Keap1 expression. In addition, lycopene can improve broiler fertility by enhancing sperm performance and reducing inflammation by modulating the levels of interleukin 1, 2, and 10 (IL-1, IL-2, and IL-10) in cases of infection. In cases of disease by aflatoxin B1 (AFB1), lycopene can modulate interferon-*γ* (IFN-*γ*), IL-1, claudin-1 (CLDN-1), and zonula occludens-1 (ZO-1). Furthermore, under the lipopolysaccharide challenge, lycopene can increase the relative weights of immune organ indices such as the bursal, spleen, and thymus.

## 1. Introduction

Animal protein consumption rapidly increases annually following the development of the social economy and population growth. Poultry products mainly fulfil this demand. Furthermore, the industry is experiencing rapid growth, particularly in developing nations [1]. Lycopene (carotenoid group) is a natural antioxidant with promising properties as a feed supplement for broiler chickens, particularly under heat-stress conditions, a significant issue in global poultry farming [2, 3]. Heat stress is correlated with the inability of animals to metabolize carotenoids, necessitating carotenoid supplementation through the diet [4]. Many fruits and vegetables provide natural carotenoids for broilers in limited amounts. Therefore, feeding these particular supplements to broilers seems appropriate for maintaining health and production.

The concentration of lycopene in fresh tomatoes (*Solanum pimpinellifolium* L) ranges from 0.594 to 03.09 mg/100 g and can be influenced by density, variety, and hue [5]. Commercial lycopene supplements are readily available although their content may vary depending on the type and formulation. Lycopene possesses notable antioxidant and prooxidant properties [6] and has been shown to mitigate the effects of reactive oxygen species (ROS) in broilers resulting from metabolic activities that generate free radicals [7]. These free radicals, unstable molecules composed of nitrogen and oxygen, can damage live cells. However, lycopene has demonstrated exceptional reactive oxygen species absorption capacity due to its conjugated structure generated from a double bond system.

Providing lycopene to broiler chickens exerts positive effects (Table 1), especially in crucial periods such as extreme temperature and infection-associated conditions [8]. The inclusion of a commercial diet supplemented with lycopene has been shown in previous research to boost yolk pigmentation and immunity in chickens throughout production [22, 23]. Therefore, this review explains the effects of lycopene in broiler chickens on their performance due to its antioxidant and immune-modulating properties.

## 2. Data Collection

Information about lycopene was gathered from various online databases such as PubMed, Google Scholar, Research Gate, Academia, and Elsevier. The keywords in the data observation were lycopene in broiler chicken, lycopene as an antioxidant, and lycopene as an immune-modulators.

## 3. Source and Biochemistry of Lycopene

Lycopene can be obtained through chemical synthesis, fermentation, or extraction from various sources, including plants, algae, fungi, and microbes [24, 25]. Natural sources of carotenoids, such as tomatoes, particularly the dark red variety and its byproducts, contain abundant lycopene. Other fruits, such as watermelon, guava, apricots, papaya, and pink grapefruit, also contain lycopene [26].

The carotenoid family is divided into subgroups based on chemical or functional features. Carotenoids are categorized as primary or secondary based on their chemical structure [2, 27]. Carotenoids are a group of isoprenoids with adaptive properties that play essential roles in plants and animals. These properties range from acting as cellular antioxidants to regulating gene expression. Therefore, their significance at the cellular and molecular levels is significant [28]. Berzelius extracted yellow pigments called xanthophylls from leaves in 1837. Tswett used chromatography to isolate and purify xanthophylls and carotenes and coined the term “carotenoids” in 1911. Between 1873 and 1927, Harsten and Willsta derived 11 g of lycopene from 75 kg of tomatoes, separating it from other carotenoids. Cámara et al. [5] later explained the chemical compositions of lycopene and other carotenoids, establishing correlations between the existence of conjugated double bonds, colour, and spectroscopic characteristics, as well as resemblances to the retinol molecule.

Lycopene belongs to the class of lipid-soluble carotenoids and is a typical isoprenoid molecule [29, 30]. The molecule contains several conjugated double bonds that give it its distinct red colour and make it easily detectable. It protects against oxidation or isomerization when exposed to oxygen, light, heat, acid, or other environmental stressors. It converts the native all-trans-isomers into cis-isomers and significantly impacts biosynthesis [31]. Due to its function as a carotenoid, lycopene can be utilized as a natural and lipid-soluble food colourant. According to previous studies, lycopene possesses anticancer, immunomodulatory, anticardiovascular, and antioxidant properties [29, 32, 33].

Lycopene is an active red carotenoid. Its molecular weight is 536.89 g/mol, containing 89.45% carbon and 10.51% hydrogen [24, 34, 35]. It can be dissolved in organic solvents, including tetrahydrofuran, hexane, chloroform, benzene, acetone, carbon disulfide, petroleum ether, and oil. Numerous efforts utilizing cutting-edge technology systems have improved its solubility and bioavailability [24, 36].

## 4. Dietary Intake Levels of Lycopene in Broiler Chicken

Poultry production is vulnerable to diseases and stress, such as pathogenic microbes and viruses, heat stress, extreme environmental conditions, and other imbalances that threaten broiler farming. According to the previous literature, a diet high in carotenoids boosted yolk pigmentation and immune protection against infection in broilers [2, 3].

According to Koutsos et al. [37], the carotenoid level in the lymphoid organs, such as the thymus and bursa of Fabricius, was higher than that in the plasma and liver of broiler chickens with a weakened immune system. The dietary supplementation with lycopene (12 mg/kg) and other compounds like fish oil and selenium enhanced birds' productivity, nutrition rate, and redox status [38].

## 5. Antioxidant Sources and Effect of Lycopene in Heat-Stressed Broilers

As a dietary supplement, exogenous antioxidants are essential since they improve poultry productivity and meat quality and maintain health by boosting the immune system and metabolism [39]. Exogenous antioxidants protect the organism from oxidative damage induced by excess ROS as the byproduct of metabolism. These radicals negatively affect the organism if they undergo oxidative stress initiated by stressful conditions, including heat exposure [7]. Lycopene supplementation can reduce free radical levels, which may be attributable to its robust antioxidant properties connected to its more remarkable singlet oxygen quenching ability, double that of alpha-carotene, and ten times that of alpha-tocopherol [40]. It eliminates free radicals by electron transfer, adduct formation, and hydrogen atom transfer [40]. Hence, lycopene simultaneously inhibits lipid peroxidation and DNA damage by inducing enzymes of the cellular antioxidant systems by triggering the transcription of antioxidant response elements [41].

According to Arain et al. [26], lycopene successfully increased levels of antioxidant enzymes (GSH-Px, SOD) and considerably decreased MDA content in muscle and blood. Moreover, adding 75 mg/kg lycopene to the broiler diet enhanced the oxidative stability of meat [9].

Lycopene therapy led to a considerable drop in liver MDA and increased liver CAT, GPX, and SOD levels in infections induced by *E. coli* [40]. Mezbani et al. [17] observed that lycopene supplementation increased the serum antioxidant state of Ross 308 broilers, as evidenced by an increase in GSH-Px activity, a decrease in MDA content (100–200 mg/kg lycopene), and an increase in CAT activity (50–200 mg/kg lycopene). Moreover, 5% lycopene supplementation boosted the antioxidant enzyme activity in the blood of heat-stressed broilers [8]. Similarly, Sahin et al. [20] indicated that increasing dietary lycopene concentration (20, 50, and 100 mg/kg food) linearly boosted serum activities of SOD and GSH-Px and lowered MDA concentration in heat-stressed broilers. Furthermore, Li et al. [42] reported that feeding broilers with tomato paste containing 10, 20, and 17 g/kg of lycopene resulted in a reduction of the levels of thiobarbituric acid-reactive substances (TBARS) and low-density lipoprotein (LDL) in broilers at weeks 2 and 4. Lycopene mediates the control of phase I and II detoxifying enzymes and gene transcription [43]. The activation of the so-called antioxidant response element boosts the production of cellular enzymes, including quinone reductase (ARE), superoxide dismutase (SOD), and glutathione S-transferase (GST) [43, 44].

A new study by Wang et al. [16] demonstrated that supplementing 1-day-old broilers with lycopene (particularly at 30 mg/kg) for 42 days significantly improved their blood antioxidant status, as evidenced by increased T-AOC, GSH-Px, and SOD activities and lowered MDA level on day 21 of the study. In addition, lycopene supplementation improved hepatic antioxidant capacity, increased GSH-Px and SOD activities, decreased MDA levels (10, 20, and 30 mg/kg lycopene), and increased GSH-Px activity (30 mg/kg lycopene) in lever.

Additional research by Sahin [45] demonstrated that increasing lycopene intake from 200 to 400 mg/kg in Ross 308 broilers decreased muscle mass. Expression of Keap1 and Nrf2 in muscle was considerably more significant in the heat-stress (HS) environment than in the thermoneutral (TN) environment. In response to oxidative stress, the Nrf2 protein is a redox-sensitive transcription factor that stimulates the production of numerous enzymes to activate the phase II detoxifying/antioxidant system [46]. Increased expression of Keap1 and decreased expression of Nrf2 in the muscle of heat-stressed broilers may result from their activation and translocation in response to oxidative stress caused by heat-stress exposure [47]. Under oxidative stress, Nrf2 dissociates from Keap1, translocates to the nucleus, and forms a heterodimer with Maf protein to stimulate the transcription of downstream genes [16]. In an experiment conducted by Wan et al. [14], the addition of 100–400 mg/kg lycopene to the diet of broilers fed AFB1-contaminated diets for 42 days dramatically decreased the hepatic activities of cytochrome P450 2A6 (CYP2A6) and P450 1A1 (CYP1A1). Lycopene has exhibited resistance against oxidative damage induced by Aflatoxin B1 (AFB1), as demonstrated by increased mRNA expression of the NRF2 signalling pathway and inhibition of Cyp450 isozymes [14, 19].

## 6. Lycopene Increase Fertility of Birds

A study by Mangiagalli et al. [48] observed that lycopene-supplemented broilers had higher fertility rates than unsupplemented broiler breeders. In addition, the enhanced sperm progressive motility and membrane integrity caused by 0.2 mM lycopene-loaded nanoliposomes therapy likely increased the quantity of functional sperm in the sperm storage tubules, boosting fertility [13]. Moreover, boosting sperm cellular features by augmenting the antioxidant system and mitochondrial activity may improve sperm function during passage through the reproductive canal [49]. This improvement in fertility was not only due to a rise in the amount of surviving spermatozoa after freeze-thawing but also to a significant improvement in their functionality. As an antioxidant in reproduction organs, lycopene administration increased TAC and GPx while lowering MDA levels in sperm [13]. These variables are associated with enhanced sperm quality. However, Puzzo et al. [21] show that the administration of lycopene did not affect the lipid peroxidation of either breast or thigh meat. Likewise, lycopene supplementation did not affect the antioxidant response of liver and kidney tissues.

## 7. Immunomodulatory Effect of Lycopene in Broilers

The effects of immunosuppressive agents on heat-stressed chickens have been demonstrated. Heat stress diminishes total antibodies and specific IgG and IgM levels during primary and secondary humoral responses. In addition, it significantly lowers the weight and size of the liver, thymus, spleen, lymph, and bursa, followed by a decrease in the number of lymphocytes in the organs [50]. In chickens, however, a loss of lymphoid tissue has been observed. Moreover, several studies have reported that heat stress can influence the number of circulating blood cells. The ratio of heterophils to lymphocytes also increases due to heat stress [50–52].

Naturally, various bird species have utilized carotenoids from fruits or leaves as potent immune-modulating antioxidants to combat cytotoxic reactive oxygen species [53] due to heat stress, which can increase susceptibility to inflammation [10]. Moreover, it induces epithelial barrier destruction, and immune cell migration contributes to the augmentation of tissue inflammation in some organs caused by NF-B as the transcription factor that plays an essential role in most chronic diseases' development, maintenance, and progression [45, 54].

As the most substantial antioxidant component in carotenoid groups, lycopene has a potential antiinflammatory effect [42, 55, 56]. Therefore, it can ameliorate intestinal injuries and increase the epithelial integrity of the digestive tract [19]. Sarker et al. [15] showed that lycopene inhibited INF-*γ* and upregulated IL-10 in the intestinal epithelium of aflatoxin B1-infected broilers. It is widely accepted that IL-10 is a strong antiinflammatory cytokine that suppresses the proinflammatory cytokines generation and dampens immune responses [57]. Moreover, lycopene lowered adhesion molecules, inflammatory genes, and proinflammatory cytokines [58]. These alterations may be attributed to the fact that cytokines are critical components of the immune system and play an essential role in defending against pathogenic bacteria [59].

Several studies have shown that lycopene supplementation can improve the immune response in broiler chickens and other animal species. For example, lycopene treatment has been reported to reduce CRP, haemoglobin, and serum TC levels in broilers infected with *E. coli* while also increasing IL-10 and phagocytic activity and decreasing IL-1 [40]. Similarly, Yonar [60] found that lycopene treatment increased oxytetracycline-suppressed phagocytic activity in rainbow trout. Lycopene's immunostimulant action may explain its ability to increase immunological parameters by boosting the synthesis of interleukin-2 (IL-2) and interferon-gamma-*γ* (INF-*γ*), which are potent activators of T cells [61]. Furthermore, lycopene may encourage the immune system to combat oxidative damage to lymphocytes' DNA [62].

Dietary lycopene at 200 mg/kg could alleviate intestinal damages caused by AFB1 by diminishing inflammatory responses and decreasing oxidative stress of the intestinal mucosa, as suggested by increased IL-the interferon-*γ* (IFN-*γ*) levels (IL-1) and increasing mRNA abundances of Cludin-1 (CLDN-1) and (ZO-1) in the jejunum [19]. CLDN1 and ZO-1 are tight junctions' most important structural and functional components and defend against pathogen growth [63]. Also, in an experiment by Wan et al. [14], the addition of 100–400 mg/kg lycopene to the feed of broilers fed AFB1-contaminated diets for 42 days decreased the blood levels of alanine aminotransferase (ALT) and aspartate transaminase (AST).

Furthermore, feeding broilers with diets supplemented at 100–400 mg/kg of lycopene lowered the activity of ACC, REBP-1, and FAS and increased the mRNA expression level of AMPK [16]. Hence, lycopene-supplemented diets in broilers could stimulate the AMPK pathway system, thereby reducing fat synthesis in the liver. Activating the AMPK pathway system may account for decreased abdominal fat formation and controlled serum lipid levels. Sun et al. [64] investigated the beneficial effects of lycopene on breeding hens exposed to lipopolysaccharide and found that feeding hen's lycopene (20–80 mg/kg) for 35 days enhanced the immune organ indices of the bursal, spleen, and thymus. However, a sizeable supplementary dose of lycopene may induce adverse effects in livestock. According to Pozzo et al. [21], supplementing the diets of Hubbard broilers with up to 500 mg/kg of lycopene lowered the weight of the bursa of Fabricius and spleen.

In addition, the increase in white blood cells, notably the proportion of lymphoid cells, and the decrease in the fraction of differentiated lymphoid cells may be attributable to the antioxidant activity of lycopene [65]. However, Pozzo et al. [21] reported that 35 days of feeding Hubbard chickens 500 mg/kg of lycopene decreased total protein, alpha globulin, gamma globulin, and albumin. In addition, an increase in apoptotic cells was identified in the spleen and bursa of Fabricius samples from lycopene-fed hens [21].

## 8. Conclusion

Lycopene supplementation can reduce free radical levels and protect the organism from oxidative damage induced by excess ROS due to heat exposure. Lycopene simultaneously inhibits lipid peroxidation and DNA damage by inducing enzymes of the cellular antioxidant defence systems by triggering the transcription of antioxidant response elements. Dietary lycopene in the feed also can increase the serum antioxidant state, lower MDA content, and boost the antioxidant enzyme activity in the blood of heat-stressed broilers. In addition, lycopene mediates the control of phase I and phase II detoxifying enzymes and gene transcription and increases fertility rates in broiler breeders.

## Figures and Tables

**Table 1:** Antioxidant effects of lycopene in the broiler's feed.

Animals	Supplemental dose	Major findings	References
Broiler	5% lycopene/kg in feed	Decreasing triglycerides, decreasing MDA concentration, and increasing SOD, GPx, ALT, AST, ALP, and lipase concentration	Hosseini-vashan, [8]
Broiler	0 and 75 mg/kg lycopene in feed	Improvement of oxidative stability	Englmaierová et al., [9]
Broiler	0, 200, 400 mg/kg lycopene in feed	Controlling the antioxidant activity of the Nrf2 and NF-kB pathways, muscle keap1, muscle Nrf2, muscle lycopene levels, and the activities of GSH-Px and SOD	Most and yates, [10]
Broiler	50–100 mg/kg lycopene in feed	Decreased the concentration of MDA	Sevcikova et al. [11]
Broiler	200–400 mg/kg	Lowered of cholesterol and MDA and increased activity of HDLC, GPx, and SOD in blood	Fathi et al., [12]
Broiler breeder rooster	0.1–0.3 mM	Improve the quality of rooster spermatozoa after thawing, increase the viability of the hatching eggs	Najafi et al., [13]
Broiler exposed aflatoxin B_1_	200 mg/kg	Enhanced GSH, GPx, and SOD activities and decreased H_2_O_2_ dan ROS concentration	Wan et al., [14]
Broiler exposed aflatoxin B_1_	200 mg/kg	Improved the redox stability by augmenting the antioxidant enzyme activity and their associated mRNA expression levels	Sarker et al., [15]
Broiler	10–30 mg/kg	Raised performance, antioxidant enzyme activities in serum and liver and upregulates genes Keap1-Nrf2	Wang et al., [16]
Broiler	50–200 mg/kg	Decreased MDA, triglyceride, cholesterol, ALP, ALT and increase catalase, GPx in blood serum	Mezbani et al., [17]
Broiler	10–20 mg/kg	Lowered LDLC, triglyceride and prevents oxidation od LDLC in liver and serum	Lee et al., [18]
Arbor acres broiler	100, 200, 400 mg/kg	Improved growth performance, BW, decrease abdominal fat, plasma lipid level, and hepatic enzymatic activity	Wan et al., [16]
Arbor acres broiler (AFB1 challenge)	100, 200, 400 mg/kg	Decreased ALT, AST, CYP2A6 activities, and hepatic CYP1A1 and increased antioxidant status	Wan et al., [14]
Arbor acres broiler (AFB1 challenge)	100, 200, 400 mg/kg	Increased growth performance, digestive enzyme, and intestinal morphology	Sarker et al., [19]
Ross 308 broiler (heat-stress conditions)	200, 400 mg/kg	Increased growth performance, serum antioxidant status, and muscle antioxidant status	Sahin et al., [20]
Hubbard broiler	500 mg/kg	Decreased growth performance, slaughter performance,	Puzzo et al., [21]

MDA: malondialdehyde; SOD: superoxide dismutase; GPx: glutathione peroxidase; ALT: alanine aminotransferase; AST: aspartate aminotransferase; ALP: alkaline phosphatase; GSH: reduced glutathione; GSSG: oxidized glutathione; T3: triiodothyronine; HDLC: high density lipoprotein cholesterol; LDLC: low-density lipoprotein cholesterol; NF-kB: nuclear factor kB; Nrf2: nuclear factor erythroid 2-related factor; keap1: Kelch-like-ECH-associated protein 1; H_2_O_2:_ hydrogen peroxide; ROS: reactive oxygen species.

## Data Availability

A search of electronic databases including PubMed, Elsevier, Research Gate, Academia, and Google Scholar yielded information about lycopene in broiler chicken. These search terms were utilized: lycopene, broiler performance, broiler production, antioxidants, and immunomodulators. In the article, the research data are presented in table format. Previous studies cited in a recent journal that is relevant to the topic of this article provided data for discussion and comparison purposes. These data are publicly accessible via the Internet. There are extensive citations in the manuscript's bibliography.
